# Management of adenoid cystic carcinoma of the head and neck: a single-institute study with over 25-year follow-up

**DOI:** 10.1186/s13005-020-00226-2

**Published:** 2020-07-02

**Authors:** Eiichi Ishida, Takenori Ogawa, Masahiro Rokugo, Tomohiko Ishikawa, Shun Wakamori, Akira Ohkoshi, Hajime Usubuchi, Kenjiro Higashi, Ryo Ishii, Ayako Nakanome, Yukio Katori

**Affiliations:** 1 Department of Otolaryngology-Head and Neck Surgery, Tohoku University Graduate School of Medicine, 1-1 Seiryo, Aoba, Sendai, Miyagi 980-8574 Japan; 2 Head and Neck Cancer Center, Tohoku University Hospital, 1-1 Seiryo, Aoba, Sendai, Miyagi 980-8574 Japan; 3 Department of Otolaryngology, Gifu University School of Medicine, 1-1 Yanagido, Gifu, Miyagi 501-1194 Japan; 4 Department of Pathology, Tohoku University Hospital, 1-1 Seiryo, Aoba, Sendai, Miyagi 980-8574 Japan

**Keywords:** Adenoid cystic carcinoma, Head and neck, Treatment, Surgery, Salvage treatment, Management, Histology, Solid type, Prognostic predictors, Lung metastasectomy

## Abstract

**Background:**

Adenoid cystic carcinoma is a rare malignant tumor arising from exocrine glands such as the major and minor salivary glands of the paranasal sinuses or the external auditory canal. Although multiple retrospective clinical studies of ACC have been reported to date, clinical questions, such as 1) long-term prognosis beyond 20 years, 2) usefulness and suitability for treatment of therapeutic interventions, 3) therapeutic goal to aim for, and 4) prognosis by recurrence sites, are still unclear.

**Methods:**

To improve understanding and management of adenoid cystic carcinoma of the head and neck (ACC), a retrospective study with 58 new ACC cases between 1991 and 2016 was performed. The median observation period was 66.8 months (range 3–316 months). The overall clinical stages were as follows: I, 6.9%; II, 25.9%; III, 19.0%; and IV, 48.2%. Histology was cribriform/tubular type (C-T type) in 62.0% and solid type in 27.5%. The main treatment strategy was definitive surgery, which was performed in 75.2% of cases.

**Results:**

Overall 10-year, 20-year, and 25-year survivals were 63.7, 27.3, and 20.0%, respectively. Similarly, disease-specific survival (DSSs) was 65.7, 51.2, and 38.4%, respectively, and disease-free survival was 25.2, 9.4, and 9.4%, respectively. Conducting surgery (HR: 0.19, 95% CI: 0.06–0.61, *p* = 0.005) and C-T type (HR: 0.32, 95% CI: 0.11–0.93, *p* = 0.036) were independent prognostic predictors of DSS. DSS was significantly prolonged after salvage surgery for both locoregional recurrence (*p* = 0.004) and lung metastatic recurrence (*p* = 0.012, vs best supportive care).

**Conclusions:**

In ACC cases, both initial surgical treatment and repetitive surgical resection of resectable recurrent lesions, including both locoregional and lung metastases, resulted in longer survival. The major goal of treatment for ACC may be long-term survival including cancer-bearing survival, resulting in either natural death or intercurrent-disease death, since judging cure of ACC is almost impossible.

**Trial registration:**

Retrospectively registered.

## Background

Adenoid cystic carcinoma of the head and neck (ACC) is a rare epithelial malignant tumor arising from exocrine glands such as the major and minor salivary glands of paranasal sinuses or the external auditory canal., and accounts for only 1% of head and neck cancers [1]. In general, while ACC takes a relatively slow clinical course, the long-term prognosis is poor because of its high frequency of local recurrence and distant metastasis [2]. The genomic hallmark of ACC is a recurrent t (6;9)(q23;p23) translocation [3] that results in a fusion between the MYB and NFIB genes [4]. The treatment consensus in ACC is resection to negative surgical margins followed by post-operative radiation (PORT) [5–7]. Whereas, patients with metastatic disease are generally incurable due to the lack of effective systemic therapies, there are a few reports of molecular pathology associated with chemoresistance such as the SWI / SNF chromatin remodeling complex [8]. Although multiple retrospective clinical studies of ACC have been reported to date [9–12], most of the data were obtained from follow-up periods of 20 years or less, and survival curves were still decreasing at the end of observation. Furthermore, it is difficult to design and conduct large prospective clinical trials because of the rareness and long course of the disease. As yet, no robust prospective clinical trial has been reported. Given this background, clinically unknown points and questions remain. Therefore, it is meaningful to conduct a retrospective clinical study with long follow-up observation, more than 20 years, in a single institution with a relatively uniform therapeutic policy. Thus, a retrospective clinical study was performed to further understand and improve management of ACC in clinical practice, by clarifying multiple clinical questions, such as 1) long-term prognosis beyond 20 years, 2) usefulness and suitability for treatment of therapeutic interventions, 3) therapeutic goal to aim for, and 4) prognosis by recurrence sites.

## Methods

### The aim, design and setting of the study

To improve understanding and management of adenoid cystic carcinoma of the head and neck (ACC) by clarifying multiple clinical questions, such as long-term prognosis beyond 20 years, usefulness and suitability for treatment of therapeutic interventions, therapeutic goal to aim for, and prognosis according to recurrence sites, 58 patients with new ACC who underwent hospitalization at Tohoku University Hospital between 1991 and 2016 were enrolled. Their medical charts, imaging examinations, and pathological prepared slides were retrospectively reviewed. A pathologist re-examined the prepared slides and confirmed no cases needed modification in the diagnoses. Imaging review was based on both enhanced MRI and CT imaging, and ^18^F-fluorodeoxyglucose-position emission tomography came into general use in combination with other studies after 2007. The UICC 7th edition was retrospectively used for both clinical TNM classification and stage in all cases except for T staging of extra-auditory canal cancer, for which the University of Pittsburgh modified staging system was used [13–15]. Sub-classifications of T stage, i.e. T4a and T4b, were combined into T4 for data analysis, because some T classifications did not include the sub-classification. The treatment strategy was as follows; initial treatments were surgery-based, i.e. surgery alone or surgery with adjuvant therapy. Most adjuvant therapy was postoperative radiation. The particle-beam radiotherapies were selected according to patients’ choice after 2004. The particle-beam treatments were options not covered by governmental health insurance throughout the targeted period, while 70% or more of the cost of all other treatments were covered. Palliative therapy was defined as neither total resection nor other aggressive treatment targeting all gross lesions. The treatment for rM1 was performed under the condition that the disease-free interval (DFI) was 12 months or more, with one or two metastases, within the range that could be resected by lobectomy, and that the residual lung function would remain sufficient. This study was approved by the Tohoku University Hospital Institutional review board (IRB; #2017–1-320).

### Statistical analysis

The Kaplan-Meier method was used for creating survival curves, and the log-rank test was used to test for significant differences between groups. Spearman’s rank correlation coefficient was used for correlation analysis, and a Cox proportional hazards model was used for multivariate analysis for prognostic factors. A significance level of 0.05 was used for each analysis, and the Bonferroni correction was used to determine the appropriate significance levels in multiple pairwise comparisons. All statistical analyses were conducted with SPSS Statics ver. 21 (SPSS, Chicago, IL, USA).

## Results

### The characteristics of participants

The patients’ characteristics are shown in Table 1. Regarding the TNM stage, while 43.1% of the cases were T4, lymph node metastasis was observed in only 7 cases (12.1%), with 2 cases of N1 and 5 cases of N2b. N2a, N2c. N3 were not observed in this study. Six cases (10.3%) showed distant metastasis, and all cases were also locoregionally advanced; either T4 or N2b. Metastatic sites were lung in 3 cases, bone in 3, and liver in 2. Each case of bone and liver metastasis also had lung metastases. Regarding the clinical stage, almost half of the cases (48.2%) were stage 4. In 43 cases (74.1%), Median postoperative radiation dose was 60 Gy (range 44–70 Gy). Only two previous cases were treated with either 40 Gy of pre-operative radiation or 60 Gy of pre-operative chemoradiation combined with cisplatin, 5-fluorouracil, and docetaxel. The particle-beam radiotherapies were selected in the recent 6 cases (10.3%), consisting of 3 cases of heavy-ion beam and 3 of proton beam therapy, and the dose range was 64.0–70.4 GyE. One case (1.7%) was treated with 70 Gy of radiation combined with selective intra-arterial chemotherapy (iaCRT) with cisplatin. Six cases with distant metastasis (cM1) and 2 cases who received best supportive care (BSC) because of their poor systemic conditions were classified in the palliation group. Of 6 cases of cM1, 2 received surgery, and 1 received iaCRT for a locoregional site. Another case received both oral chemotherapy with a fluorouracil derivative, and none of the all cM1 cases received any definitive treatments for metastatic sites.

**Table 1:** Patient characteristics (*n* = 58)

Variable	The number of patients
Median observation period (range)	66.8 months (3–316)
Median age (range)	61.5 years old (12–87)
Woman	32 (55.1%)
Primary site	
Major salivary gland	21 (36.2%)
Parotid gland	7 (12.1%)
Submandibular gland	8 (13.8%)
Sublingual gland	6 (10.3%)
Sinonasal cavity	14 (24.1%)
Maxillary sinus	11 (19.0%)
Ethmoid sinus	1 (1.7%)
Sphenoid sinus	1 (1.7%)
Nasal septum	1 (1.7%)
Pharynx	7 (12.1%)
Nasopharynx	1 (1.7%)
Oropharynx	6 (10.3%)
Oral cavity	4 (6.9%)
Palate	2 (3.5%)
Tongue	2 (3.5%)
External ear canal	6 (10.3%)
Lacrimal gland/Orbit	2 (3.5%)
Larynx	1 (1.7%)
Trachea	2 (3.5%)
Parapharyngeal space	1 (1.7%)
T classification	
T1	4 (6.9%)
T2	16 (27.6%)
T3	13 (22.4%)
T4	25 (43.1%)
N classification	
N0	51 (87.9%)
N1	2 (3.4%)
N2^a^	5 (8.6%)
N3	0 (0%)
M classification	
M0	52 (89.7%)
M1	6 (10.3%)
Overall clinical stage	
I	4 (6.9%)
II	15 (25.9%)
III	11 (19.0%)
IV	28 (48.2%)
Initial treatment	
Surgery alone	18 (32.1%)
Surgery + adjuvant therapy	25 (43.1%)
Particle-beam radiation	6 (10.3%)
Chemoradiation	1 (1.7%)
Palliation	8 (13.7%)
Surgical-margin status	
Positive (0 mm) or close to margin (< 5 mm)	28 (48.2%)
Negative (≥5 mm)	10 (17.2%)
No surgery or unknown	20 (34.5%)
Histologic pattern	
Cribriform and/or tubular	36 (62.0%)
Solid	16 (27.5%)
unknown	6 (10.3%)
Perineural invasion	
(−)	8 (13.8%)
(+)	24 (41.4%)
unknown	26 (44.8%)

^a^All N2 cases were N2b

### Survival rates and clinical outcomes

**Fig. 1:**
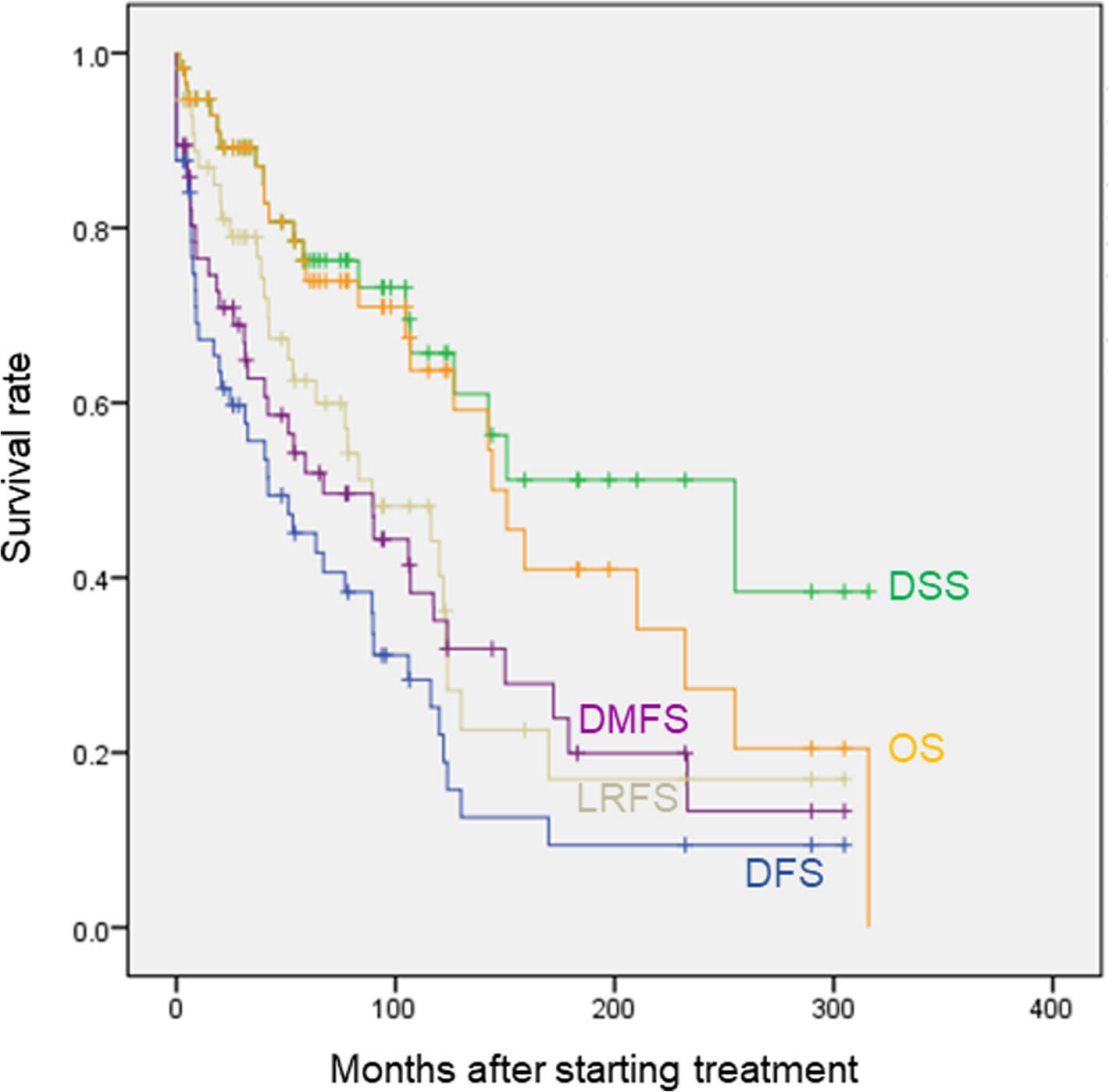
Overall survival (OS), disease-specific survival (DSS), locoregional recurrence-free survival (LRFS), distant metastasis-free survival (DMFS), and disease-free survival (DFS) of all ACC cases. Kaplan-Meier method was adopted for creating each survival curve

Overall survival (OS), disease-specific survival (DSS), locoregional recurrence-free survival (LRFS), distant metastasis-free survival (DMFS), and disease-free survival (DFS) of the whole population are shown in Fig. 1. Five-year, 10-year, 15-year, 20-year, and 25-year OS rates were 74.0, 63.7, 41.0, 27.3, and 20.0%, respectively. Similarly, DSS rates were 76.3, 65.7, 51.2, 51.2, and 38.4%, respectively; LRFS rates were 62.5, 44.2, 16.9, 16.9, and 16.9%, respectively; DMFS rates were 52.0, 35.1, 19.9, 13.3, and 13.3%, respectively; and DFS rates were 45.1, 25.2, 9.4, 9.4, and 9.4%, respectively. OS and DSS almost overlapped for more than 10 years after starting observation; the difference began to increase beyond 5% after 144.1 months (12.0 years), and it finally became 18.4% at 300 months (25 years). Of all 7 cases (12.0%) of intercurrent-disease deaths observed during the observation period, 5 died of other cancers (3 were ACC-tumor bearing), and 2 died of pneumonia (1 was ACC-tumor bearing). After the longest disease-free interval was observed at the time point of 169.9 months (14.2 years), the DFS reached a plateau, and the eventual 25-year DFS was 9.4%. On the other hand, 25-year DSS was 38.4%, which differed largely from DFS. Of all the 10 long-term-surviving cases beyond 12.0 years, when the difference between the DSS and OS started to be observed, 1 died of ACC (10%), 5 were alive (50%, 3 were ACC-tumor bearing), and 4 died of intercurrent disease (40%, 2 were ACC-tumor bearing). While intercurrent-disease deaths increased as time advanced, 60% of long-term surviving cases were eventually in an ACC tumor-bearing state.

### Prognostic factors

**Fig. 2:**
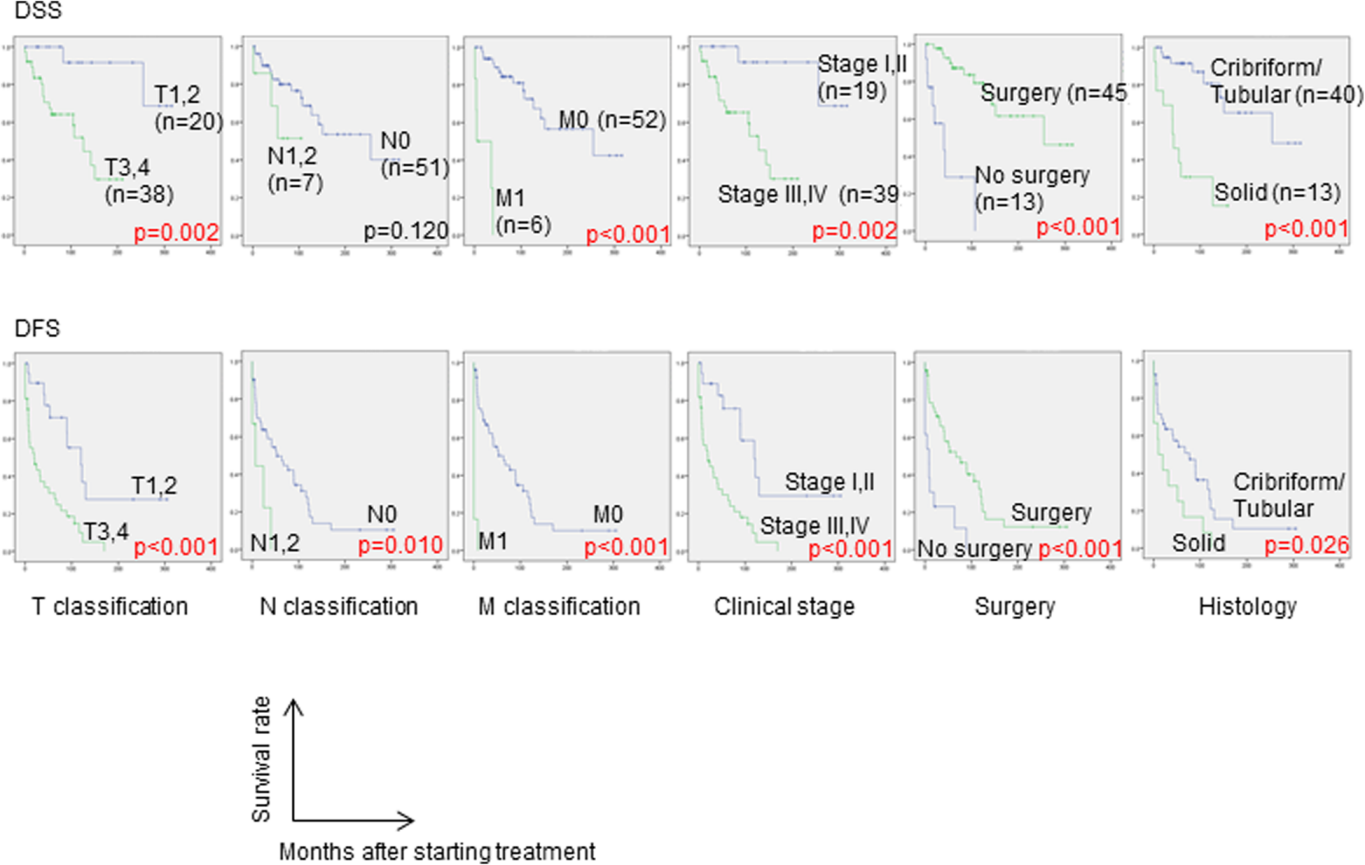
Survival curves of disease-specific survival (DSS) and disease-free survival (DFS). Kaplan-Meier method for creating each survival curve and Log-rank test for significance test between groups were adopted, respectively

Survival curves of DSS and DFS, and univariate-analysis results according to each clinicopathological factor are shown in Fig. 2. Early staged cases in either each clinical TNM classification or clinical stage showed significantly better prognosis for both DSS and DFS, except for the clinical N classification for DSS. Clinical stage correlated well with prognosis and was a comprehensively quite effective classification in ACC as well, though ACC has a wide variety of primary sites and takes different metastatic or spreading patterns from squamous cell carcinoma, which is the main histology of head and neck cancer. Regarding the initial treatment, both DSS and DFS of the surgery group (*n* = 45; 19 cases of surgery alone and 26 of surgery with adjuvant treatment) were significantly better than those of the non-surgery group (*n* = 13). No significant difference was observed between the surgery alone group and the surgery with adjuvant treatment group (data not shown). Regarding histological type, C-T type showed significantly better DSS and DFS than the solid type. No significant differences were observed in DSS and DFS according to either primary site, surgical-margin status, or perineural invasion status (data not shown).

Multivariate analyses of clinical stage, presence or absence of surgery, and histological type, which were the prognostic factors of DSS on univariate analyses, are shown in Table 2. Surgery (HR: 0.19, 95% CI: 0.06–0.61, *p* = 0.005) and C-T type of histology (HR: 0.32, 95% CI: 0.11–0.93, *p* = 0.036) were independent prognostic factors of DSS, which reduced the risk of disease-specific mortality.

**Table 2:** Prognostic factors of disease-specific survival. Multivariate analyses with variables of clinical stage, presence or absence of surgery, and histological type were performed. Conducting surgery and cribriform and/or tubular type of histology were independent prognostic factors, reducing 81 and 68% of risk of disease specific death, respectively, while early clinical stage was not independent prognostic factor. Log-rank test for univariate analysis and Cox proportional hazard model for multivariate analysis were adopted

Variable	Univariate-analysis	Multivariate analysis	
	*p* value	Hazard ratio (95%CI)	*p* value
Clinical stage	0.002^**^	0.239 (0.028–2.477)	0.191
Surgery	< 0.001^***^	0.189 (0.059–0.607)	0.005^**^
Histology	< 0.001^***^	0.316 (0.108–0.930)	0.036^*^

*** represents significance at *p* < 0.001, ** at *p* < 0.01, and * at *p* < 0.05 level

**Fig. 3:**
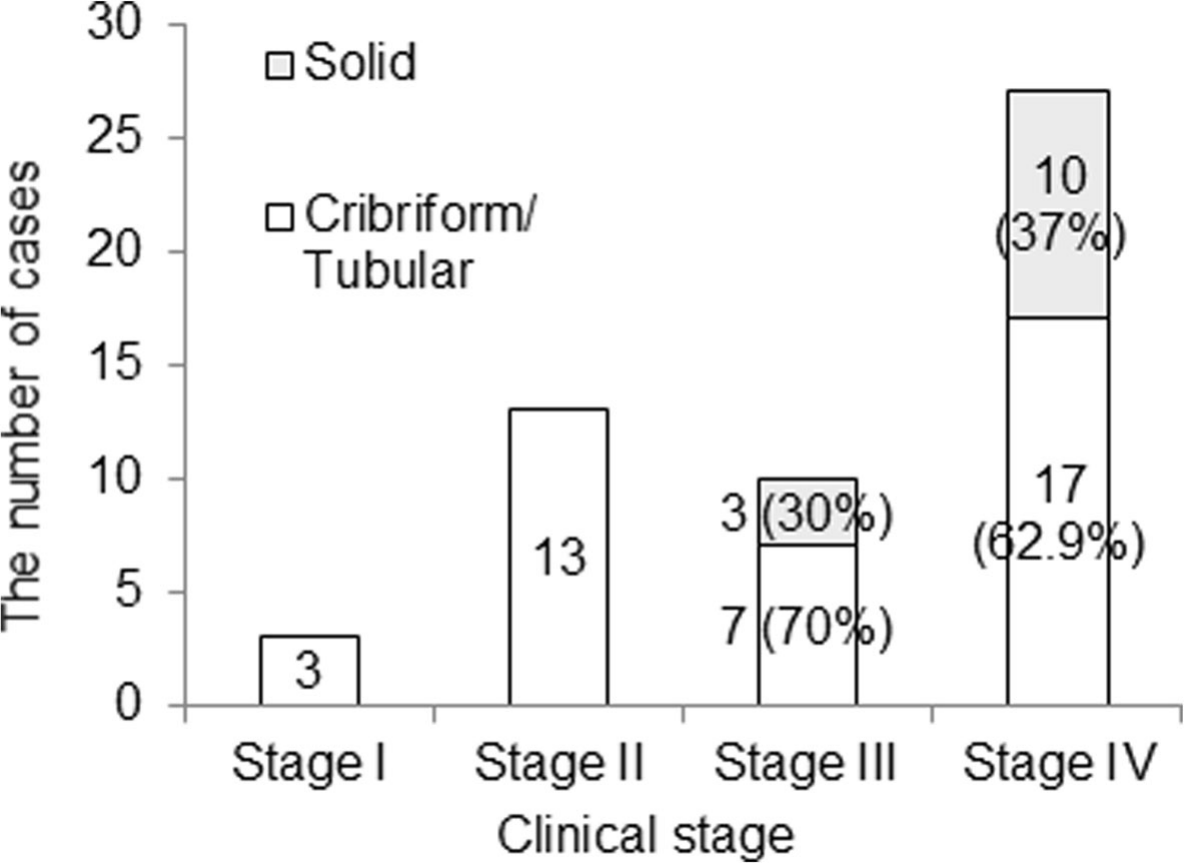
Correlations between solid type of histology and clinical stage. Solid type was observed in only advanced staged cases. The more clinical stage advanced, the higher percentage of solid type became

In order to investigate further clinical features of solid type histology, correlations between solid type and each clinicopathological factor were analyzed. Solid type was observed in neither clinical stage I nor II, while it was observed in 3 cases (30.0%) of stage III and 10 cases (37.0%) of stage IV (Fig. 3). Moreover, solid type was seen in 4 (66.6%) of 6 cases of cM1 (data not shown). Correlation analysis showed a significant correlation between histology and both clinical TNM classification and stage (Table 3). These results indicate the association between solid type and advanced stage at the initial visit. The association between solid type and recurrence was also evaluated. The analysis showed no significant difference in LRFS on univariate analysis between solid type and C-T type, but there was in distant metastatic recurrence-free survival (Supplemental Figure 1), though solid type was not an independent predictor on multivariate analysis (data not shown).

**Table 3:** Correlations between solid type of histology and clinical stage**.** Spearman’s rank correlation coefficient was adopted for analyzing correlation between histology and clinical stage

Variable1	Variable2	Correlation coefficient	p value	Significance level
Clinical stage (I,II/ III,IV)	Histology (C-T/Solid)	0.354	0.010^*^	1%
T classification (T1,2/T3,4)	Histology (C-T/Solid)	0.376	0.006^**^	1%
N classification (N0/N1,2)	Histology (C-T/Solid)	0.421	0.002^**^	1%
M classification (M0/M1)	Histology (C-T/Solid)	0.281	0.044^*^	5%

** represents significance at *p* < 0.01, and * at *p* < 0.05 level

### Recurrence and salvage treatments

Throughout the observation period, 35 (70.0%) of 50 cases who underwent radical treatments recurred. In the study of the first recurrent sites, 15 (30.3%) cases had locoregional recurrence (2 were both local and regional, and 2 were regional alone), 18 (36.0%) had distant metastatic recurrence, and 2 (4.0%) had both local and distant metastatic recurrence. Throughout the observation period, overall locoregional recurrence was observed in 24 cases (48.0%), and overall distant metastatic recurrence was seen in 28 cases (56.0%). Major first distant metastatic sites of the 34 cases that showed distant metastasis throughout the observation period were lung in 27 cases (79.4%), bone in 6 (17.6%), and liver in 4 (11.7%). Lung metastasis was also observed simultaneously in 1 case of bone metastasis and 3 cases of liver metastasis. The overall distant metastatic sites observed throughout the observation period showed only a slight increase in lung metastasis up to 29 cases (85.2%), but a large increase in bone up to 10 cases (29.4%) and in liver up to 9 cases (26.4%). Lung was the most common first distant metastatic site, followed by bone and liver, and both bone and liver metastases tended to increase over time.

**Fig. 4:**
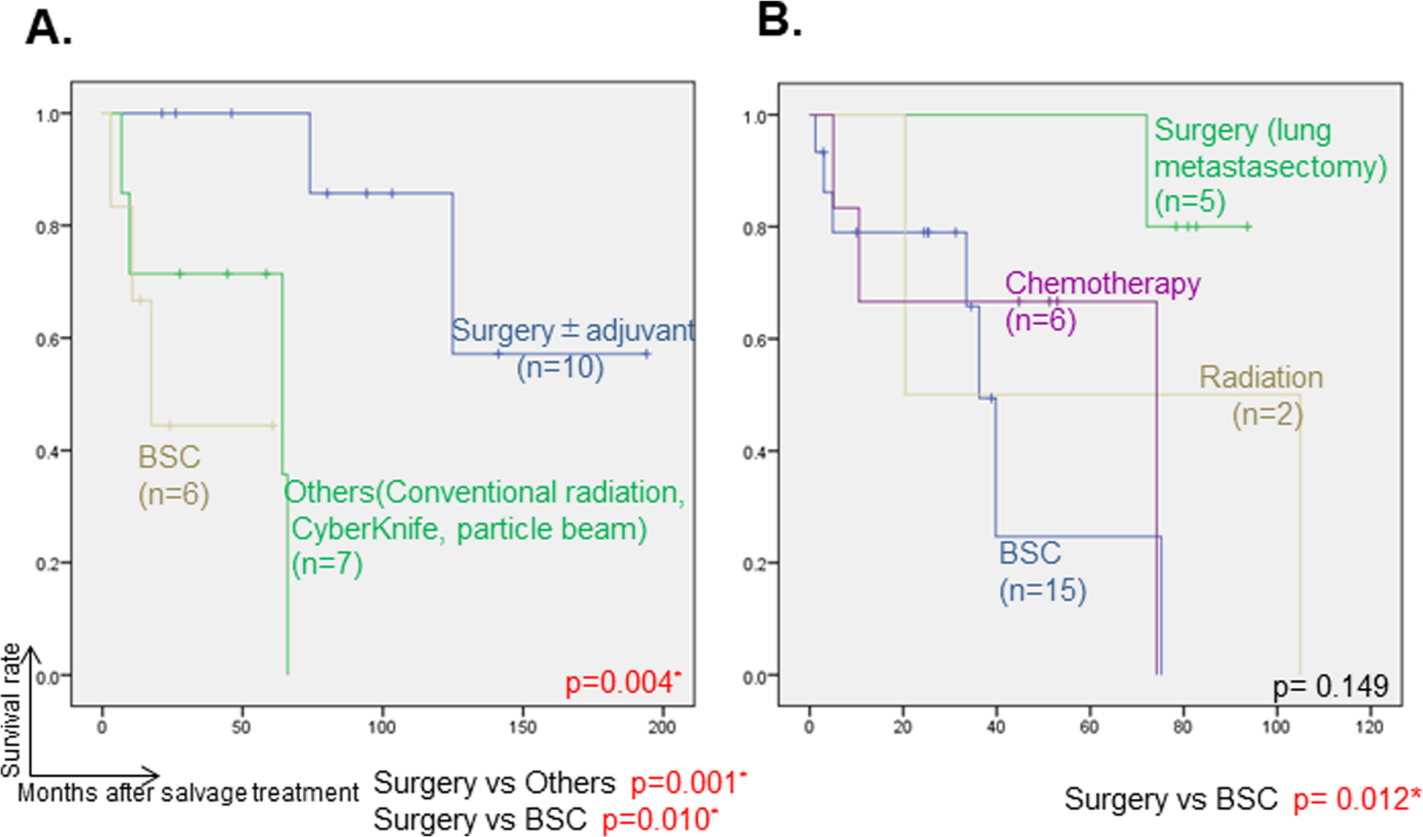
Disease-specific survival (DSS) after salvage treatment for locoregional recurrence were shown in (**a**), and for lung metastatic recurrence were shown in (**b**). Kaplan-Meier method for creating each survival curve and Log-rank test for significance test between groups were adopted, respectively. Significance level of 0.05 was used for comparison of all groups, and the Bonferroni correction was adopted to determine the proper significance levels in multiple pairwise comparisons. * represents significant *p* value. Abbreviations: DSS; disease-specific survival, Tx; therapy, BSC; best supportive care

Salvage treatments for initial locoregional recurrence in 24 cases of overall locoregional recurrence were as follows: surgery in 10 cases (3 were with postoperative (chemo-) radiotherapy), particle-beam therapy in 4 (2 carbon beam and 2 proton beam), (chemo-) radiotherapy in 3, CyberKnife in 1, and BSC in 7. DSS after salvage treatment was significantly better in the salvage-surgery group than in the other-treatment groups (*p* = 0.004, Fig. 4a).

Thirty-three cases showed distant metastatic recurrence throughout the observation period, and the salvage treatments were as follows: surgery in 6 cases (5 lung metastasectomies, including one case who underwent lung metastasectomy twice); radiation-related therapy including conventional X-ray radiotherapy, CyberKnife, and proton beam in 6; chemotherapy in 6; and BSC in 15. The median DFI in 5 patients who underwent lung metastasectomy was 43 months (range 24-122 months). In 5 patients who underwent lung metasectomy, postoperative respiratory function, swallowing, and speech function did not change from before surgery. There was no significant difference in DSS after salvage treatments for distant metastases among each salvage-treatment group (data not shown). However, for the cases of lung metastasis only, DSS was significantly better after salvage surgery, i.e., lung-metastasectomy group, than in the BSC groups (*p* = 0.012, Fig. 4b). DSS was also assessed after distant metastasis according to recurrence sites, and DSS was found to be significantly worse after either liver or bone metastasis than after lung metastasis (*p* = 0.002 and 0.006, respectively Supplemental Figure 2).

## Discussion

Although ACC is recognized to have a poor long-term prognosis, there are few reports showing long-term survival rates exceeding 20 years, and there still exist many uncertain clinical questions because of its rarity and its extraordinarily long clinical course. The present study found 25-year OS, DSS, and DFS rates of 20.0, 38.4, and 9.4%, respectively. Of the cases observed for more than 12.0 years when the difference between OS and DSS appeared, 60% of cases were finally in an ACC-cancer-bearing state regardless of whether they were alive or dead, and 30% were alive with ACC-cancer-bearing at the end of the observation period, which indicates the possibility of further decline of DSS after 25 years. Spiro et al. [2] reported that 25-year OS and DSS of ACC were 15 and 27%, respectively, and the survival curves presented in the article can be seen to show further decline of both survival rates even at 25 years when the observation period ended. While the maximum disease-free interval from initial treatment in this study was 14.2 years at the longest, longer ones of 19 years were reported in the past [2, 9]. Moreover, Jones et al. [12] reported that the local recurrence rate at 30 years was 100% in the retrospective study including the longest follow-up of 39.2 years. Considering these facts comprehensively, it is practically difficult to cure ACC, or at least impossible to judge that ACC has been cured. Coca-Pelaz et al. [6] stated the same in a systematic review article of ACC. Therefore, realistically, the major goal of treatment for ACC may be long-term survival, including cancer-bearing survival resulting in either natural death or intercurrent-disease death, rather than aiming to judge disease cure. Regarding recommended follow-up-observation period, although cost-effectiveness is a factor to be considered, unlimited follow-up may be desirable almost throughout life, because ACC takes an extraordinarily long-term course, and the long-term course beyond 30 years is not yet known. Moreover, long-term observation will result in further understanding of the disease in the future.

On multivariate analysis of prognostic factors, surgery for locoregional lesions and C-T type of histology were independent predictors of DSS. Surgical treatment has been recognized as the first-choice treatment for ACC to date [6], and the present results supported this. The effect of postoperative radiotherapy on prognosis remains controversial [11, 16–18], and in the present study, there was no significant difference between the surgery-alone group and the surgery with adjuvant therapy group. However, these results do not deny the usefulness of postoperative radiation, considering the possibility of bias that the more obvious positive surgical-margin cases might be included in the surgery with adjuvant therapy group. Regarding histological type, Perzin et al. suggested three basic patterns related to prognosis, namely tubular, cribriform, and solid [19]. They reported that the solid pattern was associated with the poorest prognosis, followed by the cribriform and tubular patterns. Our results also support this finding.

In fact, comparing the prognosis of cM1 cases (Fig. 2) and all cases showing distant metastases throughout the observation period (Supplemental Figure 2), the latter 5-year DSS was 65.1%, whereas the former was 0% (the longest survival time was 3.3 years). This large difference indicates the poor prognosis of pre-therapy distant metastatic cases.

Salvage surgery for distant metastases as well as local ones also contributed to the improvement of DSS in this study. Girelli et al. [20] examined the usefulness of lung metastasectomy for 109 cases in multiple institutions, and they reported that a DFI greater than 36 months after primary-tumor treatment and completeness of resection were the best prognostic variables of lung metastasectomy. The fact of a prognostic difference after lung metastasectomy according to the timing of the metastasis occurring might also be associated with the prognostic difference between cM1 and rM1 cases in the present study. Early distant metastasis could be attributed to solid type and/or other clinicopathological features, and they are difficult to control by treatment interventions. Because ligometastasis has also been reported to be associated with a favorable prognosis in head and neck cancer, a small number of lung metastases with long DFI may benefit from aggressive intervention even in ACC [21].

Van der Wal et al. [22] reported mean survival times after lung metastasis and after other metastases were 32.3 months and 20.6 months, respectively, and the present results also support this. Ho et al. reported that recurrent or metastatic ACC were enriched for alterations in key Notch and chromatin-remodeling genes and associated with poor prognosis [23]. Differences in these gene mutations by metastatic site may also alter prognosis.

The limitation of the present study is the small number of cases (58 cases). There was no significant difference in survival rates according to several clinicopathological factors such as primary site, perineural invasion, surgical margin, and so on, which had been reported as possible prognostic predictors in the past [1, 9, 10, 24]. In these analyses, the result was clearly affected by the weak statistical power. Although this study had one of the longest observation periods among previous reports, the observation period might not have been sufficient because the survival curves continued to decrease, and several patients were cancer bearing at final observation. Designing prospective clinical trials with longer-term follow-up is desired for further understanding of the extremely specific characteristics and course of ACC, but this may be practically difficult due to its low morbidity rate and long clinical course. Therefore, a multicenter, retrospective study with unified therapeutic strategies, sufficient case accumulation, and long-term follow-up of more than 30 years is realistic and desired.

## Conclusions

A retrospective clinical statistical analysis of 58 head and neck ACC cases with long-term follow-up was performed. While surgical intervention for locoregional lesions as initial treatment and the C-T type of histology were independent prognostic factors for DSS, the recurrence rate was extremely high, and there was a large difference between 25-year DSS and DFS. This difference was mainly attributed to salvage surgery for either locoregional recurrence or lung metastases that were resectable, indicating that not only the initial treatment but also repetitive surgical resection of resectable recurrent lesions may lead to long-term survival in ACC cases. Distant metastases seen at the first visit may be different from delayed-onset distant metastases and related to aggressive characteristics such as solid type of histology. Realistically, the major goal of treatment for ACC can be long-term survival including cancer-bearing survival resulting in either natural death or intercurrent-disease death, since it is almost impossible to judge cure of ACC.

## Supplementary information

**Additional file 1:Supplemental Figure 1.** Univariate analysis of histological type on locoregional recurrence-free survival and distant metastatic recurrence-free survival. Kaplan-Meier method for creating each survival curve and Log-rank test for significance test between groups were adopted, respectively ** represents significance at *p* < 0.01 level.

**Additional file 2:Supplemental Figure 2.** Disease-specific survival (DSS) after occurring distant metastasis. Kaplan-Meier method for creating each survival curve and Lock-rank test for significance test between groups were adopted, respectively. Significance level of 0.05 was used for comparison of all groups, and the Bonferroni correction was adopted to determine the proper significance levels in multiple pairwise comparisons. *represents significant *p* value.

## Data Availability

The datasets used and/or analysed during the current study are available from the corresponding author on reasonable request.

## Abbreviations

ACC: Adenoid cystic carcinoma of the head and neck
C-T type: Cribriform and/or tubular type
OS: Overall survival
DSS: Disease-specific survival
LRFS: Locoregional recurrence-free survival
DMFS: Distant metastasis-free survival
DFS: Disease-free survival
BSC: Best supportive care
