# Supplementary figures and images for: Management of adenoid cystic carcinoma of the head and neck: a single-institute study with over 25-year follow-up
Source: Head Face Med. 2020 Jul 2;16:14. doi: 10.1186/s13005-020-00226-2 (PMC7330995; doi:10.1186/s13005-020-00226-2)

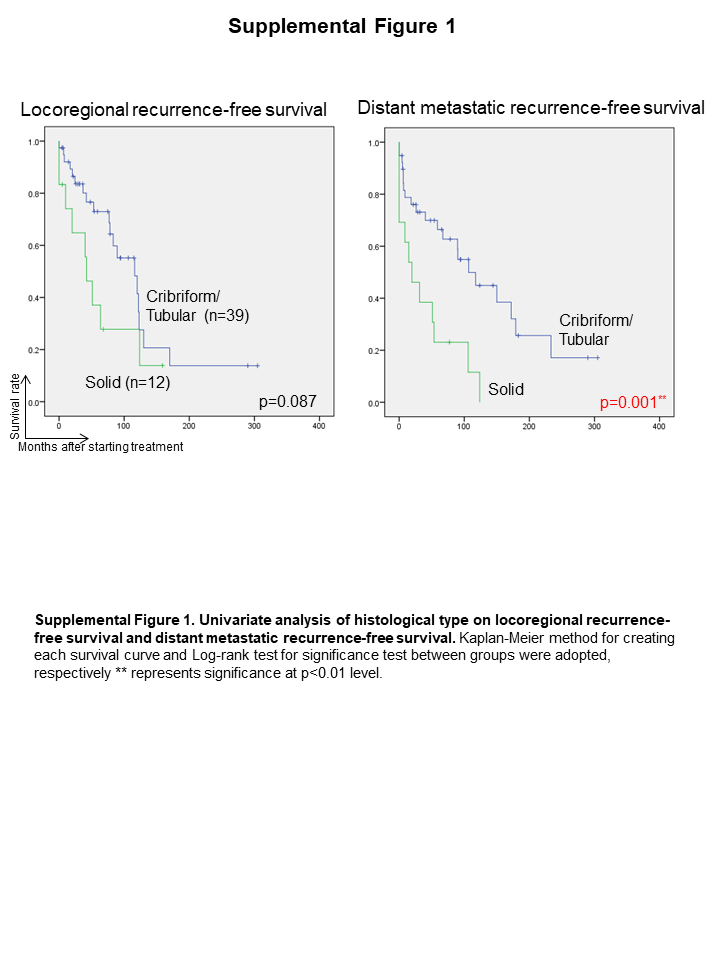

Supplement: Supplementary file 1 — Additional file 1:Supplemental Figure 1. Univariate analysis of histological type on locoregional recurrence-free survival and distant metastatic recurrence-free survival. Kaplan-Meier method for creating each survival curve and Log-rank test for significance test between groups were adopted, respectively ** represents significance at p < 0.01 level. [file 13005_2020_226_MOESM1_ESM.tif]

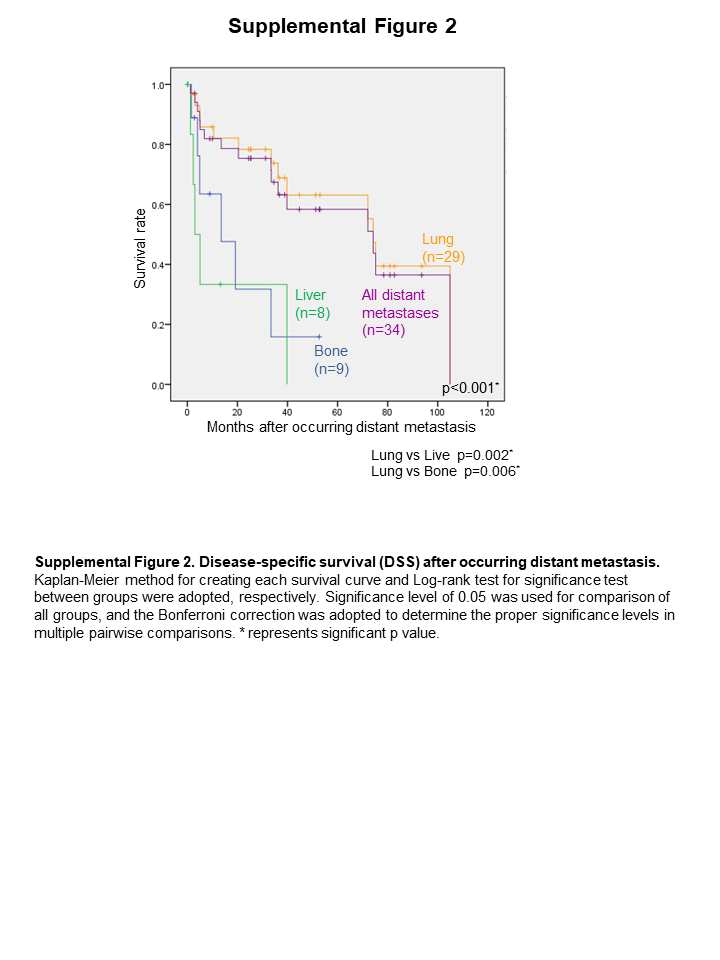

Supplement: Supplementary file 2 — Additional file 2:Supplemental Figure 2. Disease-specific survival (DSS) after occurring distant metastasis. Kaplan-Meier method for creating each survival curve and Lock-rank test for significance test between groups were adopted, respectively. Significance level of 0.05 was used for comparison of all groups, and the Bonferroni correction was adopted to determine the proper significance levels in multiple pairwise comparisons. *represents significant p value. [file 13005_2020_226_MOESM2_ESM.tif]
